# Optimized Pan-species and Speciation Duplex Real-time PCR Assays for Plasmodium Parasites Detection in Malaria Vectors

**DOI:** 10.1371/journal.pone.0052719

**Published:** 2012-12-28

**Authors:** Maurice Marcel Sandeu, Azizath Moussiliou, Nicolas Moiroux, Gilles G. Padonou, Achille Massougbodji, Vincent Corbel, Nicaise Tuikue Ndam

**Affiliations:** 1 Centre de Recherche Entomologique de Cotonou (CREC), Université d’Abomey-Calavi, Cotonou, Bénin; 2 Maladies Infectieuses et Vecteurs, Ecologie, Génétique, Evolution et Contrôle (MIVEGEC), Institut de recherche pour le développement (IRD 224), Cotonou, Bénin; 3 Centre d’Etudes et de Recherche sur le Paludisme Associé à la Grossesse et à l’Enfant (CERPAGE), Faculté des Sciences de la Santé (FSS), Cotonou, Bénin; 4 Mère et Enfant Face aux Infections Tropicales, Institut de Recherche pour le Développement (IRD 216), Cotonou, Benin; 5 PRES Sorbonne Paris Cité, Faculté de Pharmacie, Université Paris Descartes, Paris, France; Museum National d’Histoire Naturelle, France

## Abstract

**Background:**

An accurate method for detecting malaria parasites in the mosquito’s vector remains an essential component in the vector control. The Enzyme linked immunosorbent assay specific for circumsporozoite protein (ELISA-CSP) is the gold standard method for the detection of malaria parasites in the vector even if it presents some limitations. Here, we optimized multiplex real-time PCR assays to accurately detect minor populations in mixed infection with multiple *Plasmodium* species in the African malaria vectors *Anopheles gambiae* and *Anopheles funestus*.

**Methods:**

Complementary TaqMan-based real-time PCR assays that detect *Plasmodium* species using specific primers and probes were first evaluated on artificial mixtures of different targets inserted in plasmid constructs. The assays were further validated in comparison with the ELISA-CSP on 200 field caught *Anopheles gambiae* and *Anopheles funestus* mosquitoes collected in two localities in southern Benin.

**Results:**

The validation of the duplex real-time PCR assays on the plasmid mixtures demonstrated robust specificity and sensitivity for detecting distinct targets. Using a panel of mosquito specimen, the real-time PCR showed a relatively high sensitivity (88.6%) and specificity (98%), compared to ELISA-CSP as the referent standard. The agreement between both methods was “excellent” (κ = 0.8, P<0.05). The relative quantification of *Plasmodium* DNA between the two *Anopheles* species analyzed showed no significant difference (P = 0, 2). All infected mosquito samples contained *Plasmodium falciparum* DNA and mixed infections with *P. malariae* and/or *P. ovale* were observed in 18.6% and 13.6% of *An. gambiae* and *An. funestus* respectively. *Plasmodium vivax* was found in none of the mosquito samples analyzed.

**Conclusion:**

This study presents an optimized method for detecting the four *Plasmodium* species in the African malaria vectors. The study highlights substantial discordance with traditional ELISA-CSP pointing out the utility of employing an accurate molecular diagnostic tool for detecting malaria parasites in field mosquito populations.

## Introduction

Malaria remains the most prevalent parasitic disease worldwide. In 2010, an estimated 216 million malaria episodes with an estimated 655,000 deaths were reported of which more than 90% occurred in Africa [1]. Five species of the malaria parasite cause human disease. This includes *Plasmodium falciparum*, *Plasmodium malariae*, *Plasmodium ovale*, *Plasmodium vivax*, and *Plasmodium knowlesi*, which is gaining widespread recognition as a human pathogen [2]. The transmission of these malaria-causing parasites to humans is exclusively caused by *Anopheles* mosquitoes of which five species (*An. gambiae s.s.*, *An. funestus*, *An. arabiensis*, *An. moucheti* and *An. nili*) have been identified as the major malaria vectors in Africa. In southern Benin, a western African country, *An. gambiae s.s.* and *An. funestus* are the main *Plasmodium* vectors; *An. funestus* being responsible for the prolonged period of malaria transmission during the dry season [3].

Malaria in Benin is still of primary health concern among children under five and pregnant women, and motivates up to 40% of outpatient visits and 30% of hospitalizations [4]. The Malaria Control Strategy currently recommended by the WHO [5] relies on the use of the artemisinin-based combination therapy (ACT), intermittent preventive treatment during pregnancy (IPTp) and the universal distribution of Long Lasting Insecticidal Nets (LLINs). The search for an effective malaria vaccine as a supplement to the disease control strategy, remains a major aspect that holds much hope [6]. However, the success of such a vaccine, whose efforts are currently focused on *P. falciparum* malaria, raises the question of the management of mixed infections by multiple species of *Plasmodium spp.* [7].

In malaria patients, mixed species infections are common and generally under reported. A cohort study conducted on 764 children in southern Benin (Tori-Bossito) using microscopy as diagnostic tool showed the predominance of *P. falciparum* in the analyzed samples (91%), with co-infections rates involving *P. malariae* and *P. ovale* of 3% and 2%, respectively. Different patterns of mixed infections (*P. falciparum*/*P. malariae*, *P. falciparum*/*P. ovale* and *P. falciparum*/*P. ovale*/*P. malariae*) were reported in the proportions of 1.17%, 2.35%, and 0.48%, respectively [8]. As the operating characteristics of microscopy in many malaria endemic settings are known to be poor, substantial proportions of mixed-species infections can frequently be missed even by well-trained microscopists. This justifies the need for reliable alternative tool for the accurate diagnosis of malaria infection [9], [10]. In mosquito vectors, the infectious status is usually assessed by the presence/absence of *Plasmodium* sporozoites in the salivary glands. This was initially achieved by microscopic assessment of glands after the mosquito dissection. But this technique is time consuming and requires skilled staff and does not allow identification of sibling *Plasmodium species* in the sample. The development of more rapid immunological and molecular approaches such as the circumsporozoite protein enzyme linked immune-sorbent assay (ELISA-CSP) [11], [12] and PCR-based techniques rapidly got widely adopted, [13], [14]. Although ELISA-CSP seems to be relatively robust and cheap, there are potential drawbacks in using this approach. A lack of specificity has been raised as an important issue because this method does not only detect the sporozoites in the salivary glands, but can also detect CSP from other mosquito tissues [14]. An overestimation of true salivary gland infection could also result from measuring circulating CSP as this could originate from sporozoites migrating through the mosquito [15], [16]. Moreover, the ELISA-CSP technique is also subjected to an underestimation of the vector’s actual level of infection because it does not target all infecting *Plasmodium spp* in a given mosquito species [17].

In the context of the deployment of global effort towards malaria control and elimination, it is of primary importance to develop sensitive and reliable diagnostic techniques for detecting *Plasmodium spp* in both humans and mosquitoes. Recently, high-throughput assays based on real-time PCR have been developed for detecting malaria parasites in humans. These methods allow some rapid and simultaneous detection, and a quantification of several target DNAs through the use of the specific fluorophore-labeled probes [7], [18], [19]. The benefits of these methods come from very low contamination risks and high sensitivity that reaches 100 fold over the ELISA technique [14]. The use of more sensitive and effective diagnostic technique for the detection of parasites in the vectors can ensure better estimation of transmission intensity in different malaria settings.

The aim of this study was to optimize a sensitive PCR-based method that can accurately estimate mixed infection rates of *Plasmodium* species in *An. gambiae* and *An. funestus*, the main malaria vectors in Africa.

## Materials and Methods

### Study Area

The *Anopheles gambiae* mosquitoes tested in this study were collected by the team of the Centre de Recherches Entomologiques de Cotonou (CREC) Research under the framework of the President’s Malaria Initiative (PMI) program of the USAID. Mosquitoes were collected in five districts (Adjarra, Adjohoun, Dangbo, Missérété, and Sèmè) in the Ouémé department (6°34′711E – 2°31′358N) in Southern Benin.

The *Anopheles funestus* mosquitoes were collected in 3 villages in the district of Ouidah: Tokoli (6°26′57.1′′N, 2°09′36.6′′E), Lokohouè (6°24′24.2′′N, 2°10′32.1′′E) and Kindjitokpa (6°26′57.1′′N, 2°09′36.6′′E) where this species is known to be the main malaria vector [3].

The temperatures in these areas vary between 25°C and 30°C with an annual rainfall ranging from 900 mm to 1500 mm.

### Mosquito Collection and Sample Processing

Indoor and outdoor mosquito collections were conducted in two sites per village using the human landing catch technique (HLC). Collectors were hourly rotated along collection sites and/or position (indoor/outdoor). At each position, all mosquitoes caught were kept in individual tubes and in hourly bags. The collection period took place at the night between 21∶00 and 05∶00 AM.

Mosquitoes were also captured by using window traps placed in different houses in each village. The houses were selected according to the number of the people sleeping there. Traps were placed on the outside windows in each selected house from 6 PM up to 6 AM. Mosquitoes were then transferred in the cups, using a vacuum for the identification of anopheline species.

### Identification of Sibling Species and Infection Rates

All collected mosquitoes were first identified through morphological identification keys [20], [21], [22]. Female mosquitoes identified as *An. gambiae sensu lato* (Diptera: Culicidae) and *An.funestus* group were taken to CREC laboratory and stored at −20°C in Eppendorf tubes with silica gel for subsequent analyses. Heads and thoraces of *An. funestus* and *An. gambiae s.l*. were processed for detection of *P. falciparum* circumsporozoite protein (CSP) using ELISA technique as described [11], [12]. Abdomen and legs were used for DNA extraction destined to molecular identification of sibling species using polymerase chain reaction (PCR) as described previously [23], [24].

### Plasmodium Genomic DNA Samples, Plasmid Clones and DNA Standards

Mosquito’s homogenates of the head-thorax obtained from the preparation meant for ELISA-CSP (100 *Anopheles gambiae* and 100 *Anopheles funestus*) and stored at −20°C was later used for DNA extraction. Genomic DNA was extracted from the homogenates using the DNeasy® Blood & Tissue kit (Qiagen) as recommended by the manufacturer. The DNA was eluted in 100 µL and stored at −20°C. *Plasmodium* genomic DNAs of *P. vivax, P. malariae* or *P. ovale* and plasmids containing insert of the 18S gene of each of those species were kindly provided by Dr Stephanie Yanow at the Provincial Laboratory for Public Health, Edmonton, Alberta, Canada. For *P.falciparum* the 18S gene was amplified from 3D7 gDNA (MR4) using outer primers of the Nested PCR established by Snounou *et* *al*. [14], [25], and cloned into the pGEM-T vector (Promega). The insert quality was verified by sequencing. In plasmid-mixing experiments where 1.10^2^, 1.10^5^, and 1.10^7^ copies of one plasmid were mixed with similar copy numbers of the second plasmid, or 1.10^2^ copies of one plasmid were mixed with 1.10^3^, 1.10^4^, and 1.10^5^ copy numbers of the second plasmid and used as the template for the real-time PCR. Cycle threshold (CT) values were based on duplicate samples. Plasmid copy number quantification was performed by the spectrophotometric analysis. For normalization purpose, specific primers were selected and the mosquito RS7 (ribosomal protein S7) gene was amplified from gDNA of *Anopheles gambiae ss.* The purified PCR product (396 bp) was quantified by spectrophotometric analysis and used in serial dilution standard.

### Real-time PCR Assay for the Detection of Plasmodium spp in Mosquitoes

Genus-specific and species-specific primers and probes for the gene encoding the small subunit (18S) of *Plasmodium* rRNA as reported by Shokoples *et al* [7] with modification reported by Diallo *et* *al* [26] (Table 1 shows all oligonucleotide sequences used).

**Table 1:** Primers and probes used for the detection and identification of *Plasmodium* species.

Species	Primers or probe	Concn (nM)	Sequences (5′-3′)^e^
*Plasmodium* spp	Plasmo1-F primer^a^	300	GTTAAGGGAGTGAAGACGATCAGA
*Plasmodium* spp	Plasmo2-R primer^a^	300	AACCCAAAGACTTTGATTTCTCATAA
*Plasmodium* spp	Plasprobe^b^	100	VIC-TCGTAATCTTAACCATAAAC -MGBNFQ
*P*. *falciparum*	Fal-F primer^a^	300	CCGACTAGGTGTTGGATGAAAGTGTTA A
*P*. *falciparum*	Falciprobe^b^	100	FAM-TCTAAAAGTCACCTCGAAAGA-MGBNFQ
*P*. *malariae*	Mal-F primer^a^	300	CCGACTAGGTGTTGGATGATAGAGTAA A
*P*. *malariae*	Malaprobe^a^	100	FAM-CTATCTAAAAGAAACACTCAT-MGBNFQ
*P*. *ovale*	Ova-F primer^a^	300	CCGACTAGGTTTTGGATGAAAGATTTTT
*P*. *ovale*	Ovaprobe^a^	100	VIC-CGAAAGGAATTTTCTTATT-MGBNFQ
*P. vivax*	Viv-F primer^a^	300	CCGACTAGGCTTTGGATGAAAGATTTTA
*P.vivax*	Vivprobe^a^	100	VIC-AGCAATCTAAGAATAAACTCCGAAGAG AAAATTCT- TAMRA
Ribosomal protein S7	S7 FwqPCR^c^	300	5′-CATTCTGCCCAAACCGATG-3′
	S7 RvqPCR^c^	300	3′-AACGCGGTCTCTTCTGCTTG-5′
Ribosomal protein S7	S7 FwPCR^d^	300	5′-GATGGTGGTCTGCTGGTTCT-3′
	S7 RvPCR^d^	300	3′-GACACGGGAAGAGAATCGAA-3′

Footenote:

a Primers and probe sequences are as previously published [7].

b Probe sequence modified as previously published [26].

c Primers sequences are as previously published [27].

d Primers sequences are as designed in this study.

e TAMRA, 6-carboxytetramethylrhodamine; MGBNFQ, minor groove binding nonfluorescent quencher.

#### (i) Monoplex real-time PCR

A mosquito housekeeping gene (ribosomal protein S7) was amplified as an internal control to ensure that the DNA from the sample was successfully extracted and to later allow normalization when comparing different samples. PCRs were run in a final volume of 20 µl, consisting of 2 µl of DNA, 10 µl of SensiMix DNA kit (Quantace), and 300 nM of each primer. The protocol described by Dana *et* *al.* [27] allowed systematic and efficient amplification of the S7 gene in both mosquito. Reactions were run on a Rotor-Gene 6000™ (Corbett Research) using the cycling conditions of: 10 minutes at 95°C followed by 40 cycles of 95°C for 15 seconds and 60°C for 30 seconds and 72°C for 30 seconds. All amplification products were quantified using the standard range achieved with the S7 gDNA purified PCR product.

#### (ii) Duplex real-time PCR assays for Plasmodium Spp detection

Each sample was analyzed in two separate reaction tubes, containing either the genus-specific and *P. falciparum* primers and probes detection system (Plasmo/Pf) as described by Diallo *et* *al* [26] or the *P. malariae* and *P. ovale* primers/probes system (Pm/Po). Each reaction mixture contained 5 µl of DNA, 10 µl of PerfeCTa qPCR FastMix, UNG (Quanta Biosciences), 300 nM of each primer, and 100 nM of each probe in a final volume of 20 µl. Reactions underwent 40 cycles under conditions (95°C for 5 s, 60°C for 1 min). As *P. vivax* is traditionally believed to be virtually absent in West and Central Africa, the search for *P. vivax* was achieved from pooled samples. Samples were pooled into groups of 10 samples with the same amount of S7 gDNA. Five microliters of each of the pooled samples were amplified in 20 µl reaction mixtures under the same condition described above.

In order to compare parasite densities between individual samples, relative ratio was calculated by dividing the amount of *Plasmodium* DNA obtained by absolute quantification by the amount of the housekeeping DNA (S7) determined in the same sample.

### Statistical Analysis

The Cohen’s kappa coefficient (k) was used to measure inter-rater agreement between the referent ELISA-CSP and the novel real-time PCR [28]. Categorical variables were compared using Fisher’s exact test, while continuous variables were compared by the Kruskal-Wallis test. Differences were considered statistically significant when p-values were less than 0.05.

### Ethical Statements

This study was approved by the National Research Ethics Committee of Benin and the Center for Entomological Research of Cotonou (IRB00006860). All necessary permits were obtained for the described field studies. No mosquito collection was done without the approval of the head of the village, the owner and occupants of the collection house. Mosquito collectors gave their written informed consent and were treated free of charge for malaria presumed illness throughout the study.

## Results

### Mosquito Collection and Plasmodium Infection rates by ELISA-CSP

A total of 1.830 *An. gambiae s.l.* and 1.234 members of the *An. funestus* group were collected in the field and analyzed by ELISA-CSP. Fifty *An. gambiae s.s*. and twenty *An. funestus* mosquitoes were found positive to *P. falciparum*, corresponding to a prevalence of infection of 2.7% and 1.62% respectively.

In this study, 200 hundred specimens (100 *An. gambiae s.s*. and 100 *An. funestus*) were used in the evaluation of the real-time PCR assays. For *An. gambiae,* 50 *Plasmodium* infected and 50 randomly selected uninfected mosquitoes were included according to ELISA-CSP. Due to lower prevalence of *P. falciparum* infection among *An. funestus* mosquitoes, only 20 infected mosquitoes and 80 randomly selected negative were included.

### Design and Validation of the Real-time PCR Assays

The quality of the DNA extracted was checked by spectrophotometric analysis at 260 nm and 280 nm respectively, and the absence of PCR inhibitors in the preparation was confirmed by amplification of the ribosomal protein S7 in all samples; means Ct values were 24.72±2.62 for *An. gambiae* and 24.84±1.5 for *An. funestus*.

**Figure 1:**
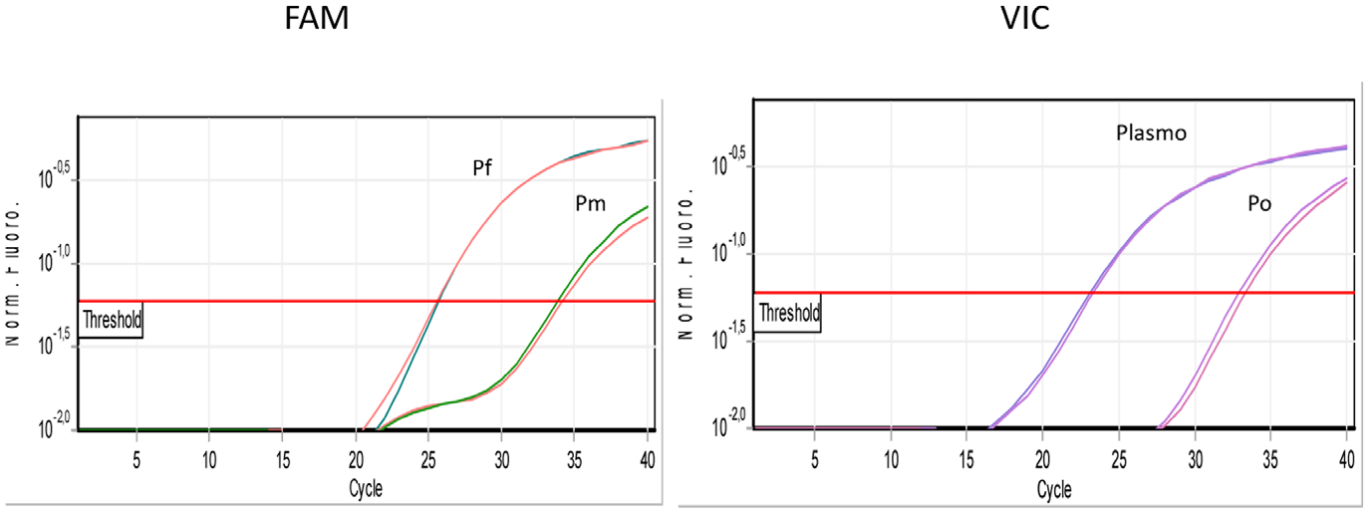
Detection of minor populations by real-time PCR in unbalanced artificial plasmids mixtures. Simultaneous detection of *Plasmodium* species DNA in a mixture of 18S specific plasmid constructs. A mixture of the three *Plasmodium* 18S rDNA targets was made with 10^5^ Pf +10^2^ Pm+10^2^ Po.

As part of the study, we first evaluated each detection system on a 10-fold dilution range of the corresponding standard (from 1.10^0^ to 1.10^10^ copies). However, the optimal linear range of the external standard curves was observed from 1.10^1^ to 1.10^10^ copies with PCR efficiencies all above 90%. Amplification of non-specific targets guided the combination of duplex assays. Evaluation of duplex assays for the simultaneous detection of *Plasmodium spp* in the same reaction was performed in plasmid mixing experiments. Results presented in Table 2 and figure 1 highlight substantial specificity and a lack of competition between the mixed oligonucleotides.

**Table 2:** Specific detection of *Plasmodium* DNA by real-time PCR in the artificial target mixtures.

	Probe FAM		Probe VIC	
Plasmids	FAL	MAL	Plasmo	OVA
Pf. 10^7^	18.15	0	18.74	0
Pf. 10^5^	24.05	0	23.91	0
Pf. 10^2^	33.84	0	33.16	0
(Po/Pm). 10^7^	0	18.2	17.86	18.83
(Po/Pm). 10^5^	0	24.66	23.52	24.66
(Po/Pm). 10^2^	0	33.45	32.05	33.90

Footnote: Validation of real-time PCR on artificial mixed targets. Plasmids constructs are: Pf, Po and Pm for *P. falciparum, P. ovale* and *P. malariae* respectively. Corresponding detection systems primers/probe are shown as FAL, MAL, OVA and Plasmo. Data are cycle threshold (Ct) values.

### Comparison of Real-time PCR Assays and ELISA-CSP

Real-time PCR analysis of the 200 mosquito homogenates revealed 65 positives and 135 negatives (Table 3). From the 70 mosquitoes (*An. gambiae* and *An. funestus*) positive for *Plasmodium falciparum* by Elisa-CSP, the presence of *Plasmodium* DNA was PCR-confirmed in 62 samples (8 samples were found negative by PCR). Among the 130 ELISA-CSP negative mosquito’s homogenates, the absence of *Plasmodium* was PCR-confirmed in 127 samples (3 samples were found positive by PCR). The real-time PCR method therefore showed relatively high values of sensitivity 88.6% (Se = 62/70*100) and specificity of 98% (Sp = 127/130*100) as compared to the ELISA-CSP here considered as the reference test. The agreement was “excellent” (κ = 0.8 and P<0.05) between real-time PCR and ELISA-CSP.

**Table 3:** Comparison of real-time PCR and ELISA-CSP for *Plasmodium spp* detection in a blind panel of mosquito samples.

Mosquito species		Real-time PCR positive	Real-time PCR negative	Total
*An. gambiae*	Elisa-CSP positive	42	8	50
	Elisa-CSP negative	1	49	50
*An. funestus*	Elisa-CSP positive	20	0	20
	Elisa-CSP negative	2	78	80

Footenote: A total of 43 and 22 positive samples were detected by real-time PCR in *An. gambiae* and *An. funestus* respectively. Real-time PCR did not confirm the ELISA-CSP results on 11 samples (9 in *An. gambiae* and 2 in *An. funestus*). ELISA –CSP was considered as a gold standard and the agreement between the two methods was “excellent” (κ = 0.8 and P<0.05 by Chi-square test).

### Prevalence and Co-infection Rates with Plasmodium spp in Mosquitoes

**Figure 2:**
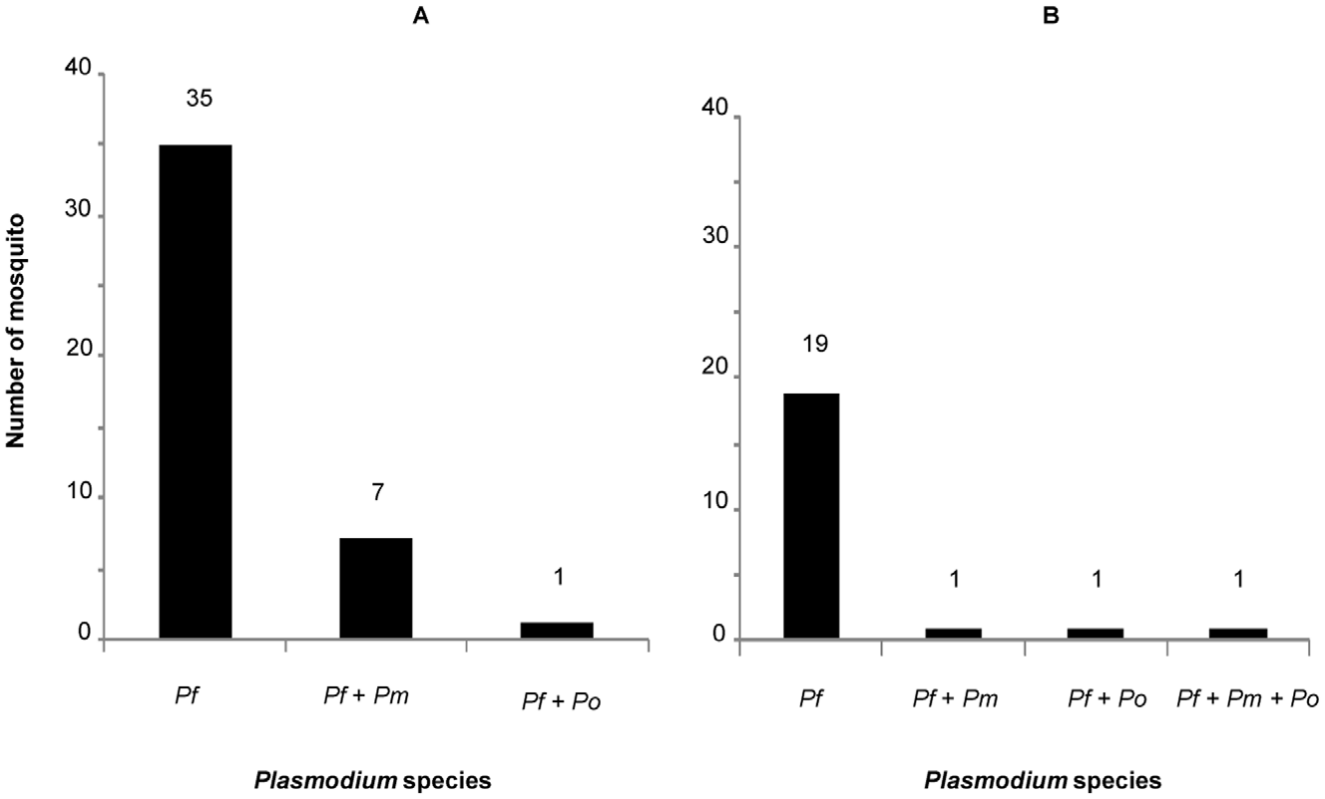
Prevalence of co-infection of *Plasmodium spp* in mosquitoes (*An. gambiae* and *An. funestus*) by Real-time PCR. The figure (A) shows results of speciation analysis of 43 positive samples of *An. gambiae* ss by qPCR. Of the 43 positive samples, 35 were infected by *P. falciparum* only (81%), 7 samples showed mixed infection with *P. falciparum* and *P. malariae* (16%), and a mixed infection with *P. falciparum* and *P.ovale* was observed in 1 sample (2%). The figure (B) shows results of analysis of 22 positive samples of *An. gambiae* ss by qPCR. Among the 22 positive samples, mono infection with *P. falciparum* was found in 19 samples (86%), 1 sample showed mixed infection with *P. falciparum* and *P. malariae* (4.5%), mixed infection with *P. falciparum* and *P. ovale* was observed in 1 sample (4.5%), and in 1 sample mixed infection with 3 species (*P. falciparum*, *P. malariae* and *P. ovale)* was noted (4. 5%).

The infection rates with *Plasmodium spp* are shown in Figure 2. The speciation by real-time PCR of the 43 PCR positive *An. gambiae s.s*. and 22 PCR positive *An. funestus* revealed the presence of *P. falciparum* in all samples (100%). While mono infections with only *P. falciparum* were found in 81.4% and 86.4% of infected *An. gambiae* and *An. funestus* respectively, mixed infections with multiple *Plasmodium* species were detected in 18.6% and 13.6% of the respective samples. Of particular remark, co-infections in the *An. gambiae* specimen predominantly involved *P. falciparum* and *P. malariae* (detected in 16.2% of samples) while mixed infections with *P. falciparum* and *P. ovale* were detected in 2.4% of the samples. In *An. funestus,* mixed infections involving *P. falciparum* and *P. malariae* or *P. falciparum* and *P. ovale* were each found in 4.5% of the samples and one samples harboured all 3 species (*P. falciparum*/*P. malariae*/*P. ovale*).

The comparison between co-infection rates involving *P. falciparum* and *P. malariae* between *An. gambiae s.s* (16.2%) and *An. funestus* (9%) showed no significant difference (Fisher’s exact test, P = 0.7631). *P. vivax* was not detected in any sample.

### Absolute and Relative Quantification of Plasmodium spp DNA in Mosquitoes

Absolute quantification of all positives specimen was done using the standard curve generated on *Plasmodium falciparum*-specific18S DNA plasmid dilutions amplified in each run with the Plasmo/Pf detection system. The standard curve was generated on the 10-fold dilution of *Plasmodium falciparum*-specific plasmid range (1.10^1^ to 1.10^6^ copies in 5 µL reaction). Samples in which the target was detected at a late Ct value beyond the linearity range (36<Ct<40) were considered positive but not quantifiable. Three ranges (1.10^1^, 1.10^3^ and 1.10^6^) of *P. malaria/P. ovale* plasmid mixture were amplified consistently with the detection system Po/Pm in each run. *Plasmodium* Pan-species and all species-specific Real-time PCR yielded efficiencies (E) above 90%. Among the 43 positives samples of *An. gambiae, Plasmodium* DNA was quantified in 39 samples (absolute copy number ranging from 10 to 913 copies, with the median, 158; [IQR = 93.65–259.8]) and in *An. funestus, Plasmodium* DNA was quantified in 19 samples of the 22 positive samples (absolute copy number ranging from 51.6 to 816 copies with the median, 333.9; [IQR = 198.9–527.7]). At the species level, DNA target specific to *P. falciparum* was detected in all positive samples and in the cases of mixed infections with multiples Plasmodium species, *P. falciparum* DNA was always detected at earlier Ct value indicating that it represented the dominant species.

**Figure 3:**
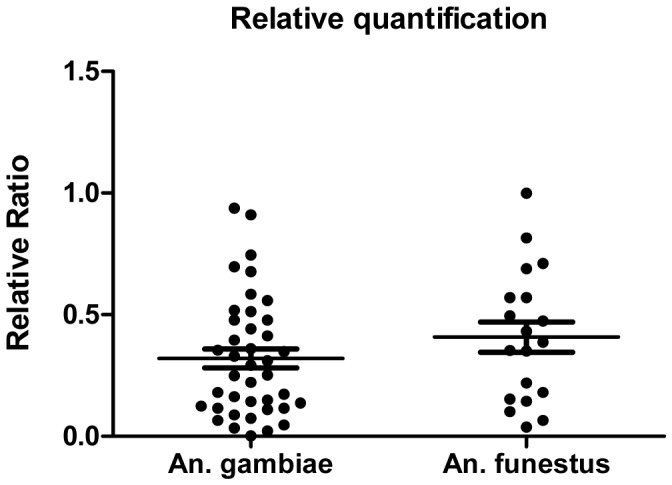
Absolute and relative quantification of *Plasmodium* DNA in mosquitoes. This figure shows a not significant difference was observed in the *P. falciparum* densities between the two *Anopheles* species (P-value = 0, 2197).

The PCR amplification of ribosomal protein S7 gene (E = 99%) allowed the estimation of the amount of mosquito DNA in each reaction. Normalization of the amount of *Plasmodium* DNA on the amount of mosquito DNA showed no difference in parasite load observed between the infected *An. gambiae* and *An. funestus* studied (Kruskall-Wallis test, P = 0.2197) (Figure 3).

## Discussion

In the context of malaria elimination (eradication) policy, it is essential to develop reliable diagnostic techniques for detecting *Plasmodium spp* infections in humans and in the vector. The aim of this study was to optimize a high-throughput sensitive and specific real-time PCR assay to detect and quantify *Plasmodium* infections in malaria vectors in Benin. Here, we used the same region of DNA encoding the small subunit of the18S rRNA to redefine the optimal multiplex PCR assays based on allele-specific primers/probe systems previously reported by Shokoples et al. [7]. Minor sequence modifications and labeling were made on the probes used by Shokoples et al. [7] to increase specificity and adapt the method to a duplex-based detection system [26]. The genes encoding the small subunit of the18S rRNA exist in several copies (7) in the Plasmodium genome. One of the major advantages of the method previously reported by Shokoples et al. [7] over other approaches [13], [18], [19], is that the primers designed target all copies thus increasing the sensitivity of the reaction. The use of species-specific oligonucleotides that could accurately detect all four malaria-causing *Plasmodium* species (Pf, Pm, Po and Pv) without significant competition between the oligonucleotides designed for the different templates was one of the major advantages of this approach. Here, the multiplexing of the reaction was optimized for the simultaneous detection of the four *Plasmodium* species at a time in two reaction tubes. This method was tested on plasmid preparations and showed good amplification efficiencies (E>90%). We also noticed a good sensitivity with the ability of detecting and quantifying down to 10 copies of Plasmodium 18S rDNA in 5 µL DNA used per reaction, meaning that at least 200 copies, approximately 30 sporozoites, are necessary in DNA preparation for a positive reaction to be quantified. Targets copy number detected below this threshold were still considered positive but unquantifiable as this falls outside the linearity range of external standards. The specificity of the real-time PCR was demonstrated by the absence of cross-reactivity between different primer-probe systems on artificial mixtures of plasmid preparations. The analytical sensitivity of the assays for *P. malariae*, *P. ovale* and *P. vivax* as the minor species in cases of mixed infection with *P. falciparum* showed, like in the data reported by Shokoples et al. [7] that we could reproducibly detect minors populations at a greater fold down to 1∶1000 ratio. This performance was optimized by the formulation of the multiplexing that we have defined (Plasmo/Pf and Pm/Po). With these modifications, we implemented this assay as a confirmatory test for malaria species identification in anopheline vectors (*An. gambiae* and *An. funestus*). *Plasmodium* DNA was consistently amplified from frozen mosquito homogenates initially prepared for ELISA. This suggests that parasite target DNA will likely remain detectable by PCR in mosquito homogenates for longer periods, from the time they are stored at −20°C. This preservation condition of field-collections for subsequent target PCR-detection of *Plasmodium* DNA is highly amenable to field work and does not seem to promote biological degradation processes which are favored by the release of nucleases after grinding. In comparison with traditional ELISA-CSP; the real-time PCR assay was more useful for the identification of *Plasmodium* species in the vectors. From the 70 positive mosquitoes for *P. falciparum* by ELISA-CSP the presence of *Plasmodium* could be PCR-confirmed in 62 samples. Of important diagnostic significance, 11 samples were misdiagnosed by ELISA-CSP. Among these, 8 samples that were positive by ELISA-CSP were not confirmed by real-time PCR. The absence of *Plasmodium* DNA was further ascertained in those samples by using the conventional nested PCR described by Snounou et al [14]. These results are concordant with the hypothesis that ELISA-CSP may be compromised by overdiagnosis and poor specificity due to circulating antigens originating from the rupture of oocysts [15] or from other non-malaria antigens [29]. This phenomenon has been highlighted in other studies when testing the plasma fractions of pig and bovine blood in Thailand [30]. In Senegal, false positive results were also associated with bovine and/or sheep blood meals when testing *An. gambiae s.l.* for the presence of *P. malariae* and *P. ovale* [31]. In most studies where false positive ELISA results were reported in mosquitoes [31], [32], [33], [34], only head and thoraces were used for ELISA assays hence excluding the possible contamination with oocyst-sporozoites present in the abdomen.

In this study, the real-time PCR identified 3 new cases of positive mosquitoes that have been missed by the ELISA-CSP. The apparent discrepancy between the two diagnostic methods on the positives samples may be due to the detection limit of ELISA-CSP. Actually ELISA-CSP has a detection limit of 250 sporozoites in 50 µl [35], while the detection limit for PCR in salivary glands was previously estimated at 10 sporozoites [13], this being lower with the real-time PCR used in this study.

The analysis of all *Plasmodium* positive samples of *An. gambiae* s.s. and *An. funestus* by real-time PCR revealed the presence of *P. falciparum* in all the samples. This is not surprising because *P. falciparum* is the most prevalent malaria parasite in west and central Africa. The relative quantification applied to normalize the copy number of *Plasmodium* target detected to that of the amount of mosquito DNA showed that parasite densities could vary between individual mosquitoes but were on average similar between infected specimens of either *An. gambiae* or *An. funestus.* These findings confirm the trend that *An. funestus* is an important vector of malaria parasites in Benin [3].

Following the high specificity of real-time PCR technique demonstrated in target mixing experiments, the assay allowed us to identify other *Plasmodium* species alongside the dominant species *Plasmodium falciparum*. These species occurring mainly as minor populations in cases of coinfection. The rate of mixed infections of 18.6% and 13.6% in *An. gambiae* and *An. funestus* respectively appeared greater than what is described in human populations living in southern Benin. Actually the proportion of co-infection with *P. ovale* and *P. malariae* in humans was only estimated to 5% [8]. Though this difference is potentially due to the methods used in the two studies (microscopy and real-time PCR), a recent analyses of human specimen in Southern Benin with the same real-time PCR technique revealed less frequency of co-infections rate than what we observed in mosquitoes (Tuikue Ndam et al., Unpublished data). This suggests the existence of other factors, including the immunity that may affect the diversity of *Plasmodium* infection in humans and mosquito. *Plasmodium vivax* was detected by real-time PCR in none of the specimens studied. This was not surprising as this species is known to be very rare in west and central Africa.

### Conclusion

This study reports the analytical validation of new real-time PCR assays for the detection and identification of *Plasmodium* species in the mosquito hosts. Combined with efficient DNA extraction methods, the assay has demonstrated a good analytical sensitivity in detecting mixed infections with distinct malaria-causing *Plasmodium* among the two main malaria vectors in Benin. The results suggest that the method described here is appropriate for the detection of malaria parasites in field-collected mosquito specimen. The application of this highly specific multiplex real-time PCR assay in larger and prospective studies will allow a better appreciation of the intensity of transmission and the knowledge of the movement of different *Plasmodium* species in the malaria vectors in Africa. This technique could facilitate the implementation of more basic research to address the fitness cost associated with *Plasmodium spp* infections on several life history traits including survival, behavioral and reproductive capacity of mosquito vectors.
